# The Thioredoxin System Is Regulated by the ASK-1/JNK/p38/Survivin Pathway during Germ Cell Apoptosis

**DOI:** 10.3390/molecules24183333

**Published:** 2019-09-12

**Authors:** Nora Al-Kandari, Fatemah Fadel, Farah Al-Saleh, Farah Khashab, May Al-Maghrebi

**Affiliations:** Department of Biochemistry, Faculty of Medicine, Kuwait University, Jabriyah 13110, Kuwait; norayaq@gmail.com (N.A.-K.); fatma.alshty@gmail.com (F.F.); farah.saud92@gmail.com (F.A.-S.); dashtikh91@gmail.com (F.K.)

**Keywords:** germ cell apoptosis, testicular ischemia reperfusion injury, oxidative DNA damage, thioredoxin, survivin, ASK-1, JNK, NQDI-1

## Abstract

The aim is to explore the mechanism of the apoptosis signal-regulating kinase-1 (ASK-1) signaling pathway and the involvement of the thioredoxin (Trx) system during testicular ischemia reperfusion injury (tIRI) by using ASK-1 specific inhibitor, NQDI-1. Male Sprague-Dawley rats (*n* = 36, 250–300 g) were equally divided into 3 groups: sham, tIRI, and tIRI + NQDI-1 (10 mg/kg, i.p, pre-reperfusion). For tIRI induction, the testicular cord and artery were occluded for 1 h followed by 4 h of reperfusion. Histological analyses, protein immunoexpression, biochemical assays, and real-time PCR were used to evaluate spermatogenesis, ASK-1/Trx axis expression, enzyme activities, and relative mRNA expression, respectively. During tIRI, ipsilateral testes underwent oxidative stress indicated by low levels of superoxide dismutase (SOD) and Glutathione (GSH), increased oxidative damage to lipids and DNA, and spermatogenic damage. This was associated with induced mRNA expression of pro-apoptosis genes, downregulation of antiapoptosis genes, increased caspase 3 activity and activation of the ASK-1/JNK/p38/survivin apoptosis pathway. In parallel, the expression of Trx, Trx reductase were significantly reduced, while the expression of Trx interacting protein (TXNIP) and the NADP^+^/ nicotinamide Adenine Dinucleotide phosphate (NADPH) ratio were increased. These modulations were attenuated by NQDI-1 treatment. In conclusion, the Trx system is regulated by the ASK-1/Trx/TXNIP axis to maintain cellular redox homeostasis and is linked to tIRI-induced germ cell apoptosis via the ASK-1/JNK/p38/survivin apoptosis pathway.

## 1. Introduction

Germ cell apoptosis (GCA) is one of the consequences of testicular ischemia reperfusion injury (tIRI) that underlies the etiology of testicular torsion and detorsion (TTD), a urologic emergency that affects young males [1,2]. The gold standard treatment for TT is to surgically detorse the ischemic testis to allow for blood and oxygen reperfusion. The testis is characterized by a low oxygen tension microenvironment, thus, a sudden influx of oxygenated blood will create a state of testicular oxidative stress (TOS) due to the generation of massive quantities of reactive oxygen species (ROS). Overwhelming Reactive Oxygen Species (ROS) will start to accumulate due to the incapacity of the intracellular antioxidant system to neutralize them. Under physiological conditions, the spermatozoa produces ROS by an aerobic metabolism that is required for normal sperm function, to facilitate capacitation, and to promote fertilization [3]. However, overproduction and accumulation of ROS will have adverse effects on sperm motility, fertilization competence, and viability due to oxidative damage to proteins, lipids, and nucleic acids [3]. Under such conditions, testicular germ cells and somatic cells will fail to commit to spermatogenesis and steroidogenesis, respectively, and are forced to undergo apoptosis.

The apoptosis signal-regulating kinase-1 (ASK-1) is an early response stress mitogen-activated protein kinase kinase kinase (MAPKKK) to excessive ROS accumulation and functions upstream of other kinases such as MAP3K, JNK and p38 [4]. The activity of the oxidant-sensitive ASK-1 is closely regulated by its natural inhibitor thioredoxin (Trx) [5]. Interestingly, Trx is a member of the Trx system that also includes thioredoxin reductase (TrxR) and NADPH, which is essential for maintaining the redox status of the cell and an effective antioxidant system [6]. The Trx antioxidant effects can be mediated by either interacting with Trx interacting protein (TXNIP) or as a direct antioxidant molecule [7]. Under OS, Trx becomes oxidized, detaches from ASK-1 and initiates the activation of the ASK1 signalosome [5]. Continuous activation of ASK-1 signaling will induce the activation of the mitochondria-dependent caspases, leading to activation of the intrinsic apoptosis pathway [8]. However, there is limited research investigating the cross-link between ASK-1 and the Trx system during pathological conditions and none has explored how the ASK-1-Trx axis is modulated during tIRI-induced TOS and subsequent GCA. Thus, suppression of ASK-1 activity may shed a light on its physiological interaction with Trx and its effect on prevention of TOS-induced GCA. Ethyl 2,7-dioxo-2,7-dihydro-3*H*-naphtho[1,2,3-*de*]quinoline-1-carboxylate (NQDI-1), is a specific and strong enzyme activity inhibitor for ASK-1 and not any other kinase [9]. The inhibitory action of NQDI-1 is based on its competitive ability to occupy the ATP binding site in ASK-1, act as a phosho-donor ATP substrate and prevent downstream ASK-1 phosphorylation [10]. The aim of this study is to assess the effect of NQDI-1 on tIRI-induced spermatogenic and oxidative damages, the expression of the Trx system components and the activation of the ASK-1 apoptosis signaling pathway.

## 2. Results

### 2.1. NQDI-1 Protects against Damge to Testicular Histology and Spermatogenesis

**Figure 1:**
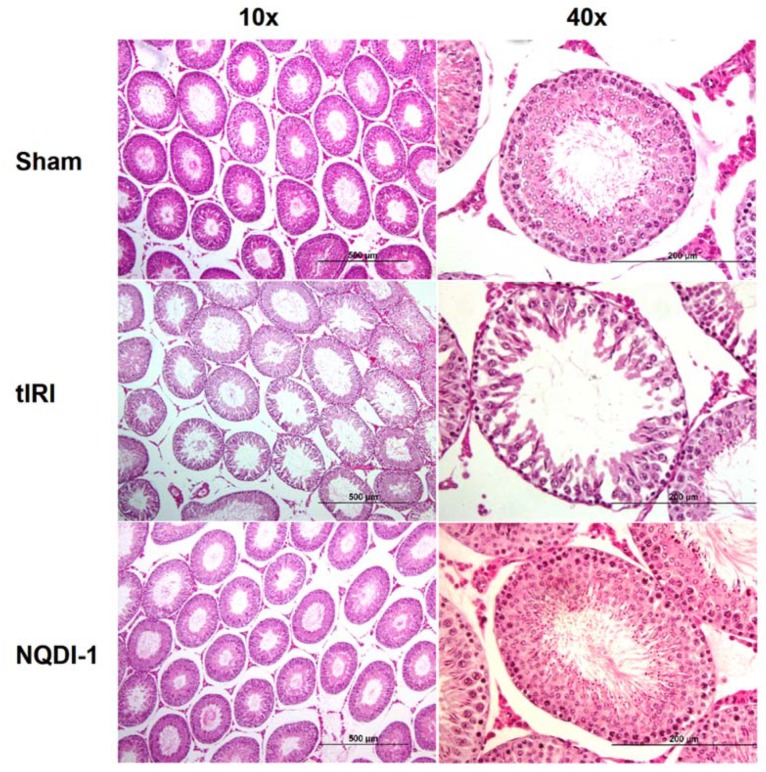
NQDI-1 protects against damage to testicular histology and spermatogenesis. hematoxylin and eosin (H&E)stained-slides were visualized under light microscopy revealing destruction in the Seminiferous Tubule (ST) structure accompanied by alterations in the germ cell layer arrangement after tIRI induction. In contrast, sham and NQDI-1 treated groups showed normal ST histology and proper cellular stages of spermatogenesis. Contralateral testes showed no significant difference between the three animal groups. NQDI-1 (10 mg/kg) was i.p. injected 30 min post ischemia. Images were taken at 10x and 40x magnification with a scale bar of 50 μm.

To evaluate damage to spermatogenesis, hematoxylin and eosin (H&E) staining was performed, slides were analyzed under light microscopy (10× and 40× magnifications), and the Johnsen score was used to assess spermatogenesis in each testis in each experimental group (Figure 1).

Among the ipsilateral testes, the sham operated rats revealed normal spermatogenesis represented by the absence of disruptive germinal layers and an intact Seminiferous Tubule (ST)structure with a Johnsen score of 9.7 ± 0.52. The tIRI-subjected rats, on the other hand, had impaired histological appearance with the presence of very few spermatocytes with no spermatids or spermatozoa present with disrupted ST structure and a low Johnsen score of 4.8 ± 0.41 (*p*-value < 0.0001 compared to sham). The NQDI-1-treated rats showed normal spermatogenic distribution and improved arrangement of germ cell layers, with a Johnson score of 8.7 ± 1.0 (*p*-value < 0.0001 compared to tIRI). Contralateral testes from all groups had a normal spermatogenesis and ST structure (Sham: 9.5 ± 0.55, tIRI: 8.8 ± 0.75 and NQDI-1: 9.7 ± 0.52; *p*-value > 0.05).

### 2.2. NQDI-1 Inhibits Testicular Oxidative Stress

**Figure 2:**
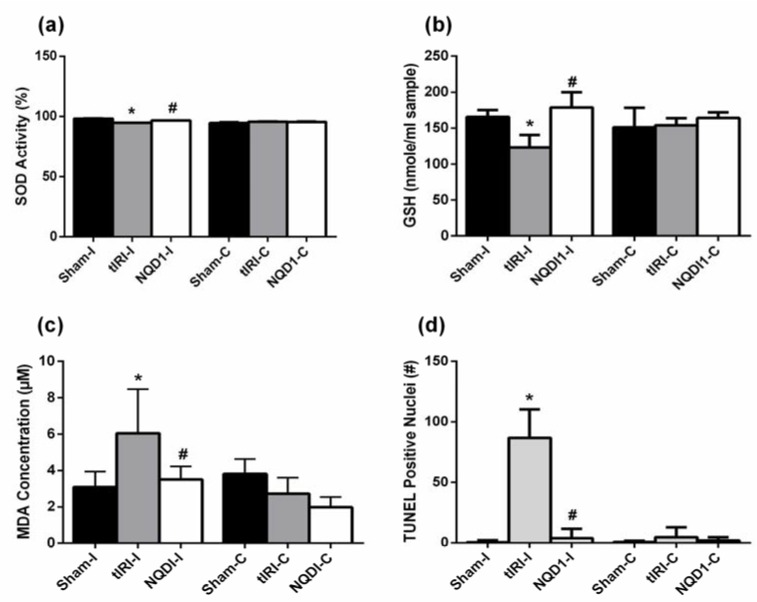
NQDI-1 inhibits testicular oxidative stress. Colorimetric assays were used to analyze (**a**) SOD activity; (**b**) GSH levels and (**c**) MDA concentration, while Terminal deoxynucleotidyl transferase dUTP Nick End Labeling (TUNEL) fluorescent staining was used to assess (**d**) DNA strand breaks. During tIRI, ipsilateral testes exhibited lower levels of the antioxidants SOD and GSH but high levels of MDA and DNA strand breaks in comparison to sham. NQDI-1 treatment attenuated testicular OS. Contralateral testes showed no significant difference between the three animal groups (*p*-value > 0.05). NQDI-1 (10 mg/kg) was i.p. injected 30 min post ischemia. Data are presented as mean ± SD (*n* = 6/group). * tIRI compared to sham and # NQDI-1 compared to tIRI. I = Ipsilateral and C = Contralateral.

To assess the oxidative status of the testis, the following parameters were measured: the antioxidants levels of superoxide dismutase (SOD)and Glutathione (GSH), the lipid peroxidation marker malonaldehyde (MDA) and oxidative DNA strand breaks (Figure 2).

During tIRI, the ipsilateral testes exhibited lower SOD enzyme activity (%) compared to sham levels (94.7 ± 0.34 vs. 98.1 ± 0.56, *p*-value < 0.0001). The NQDI treatment increased SOD activity in comparison to the tIRI group (96.7 ± 0.20 vs. 94.7 ± 0.34, *p*-value = 0.033) (Figure 2a). Contralateral testes had no significant changes between the three groups (sham = 94.6 ± 1.12, tIRI = 95.7 ± 0.526 and NQDI-1 = 95.4 ± 0.927; *p*-value > 0.05), but are similar to the SOD activity of tIRI-I. This could indicate that the obtained SOD activity, although statistically significant, might not represent biological relevance in this case.

In ipsilateral testes, low GSH concentrations (nmole/mL sample) were measured following tIRI as compared to sham (123 ± 17.3 vs. 166 ± 9.53, *p*-value = 0.0172). The GSH activity was significantly increased after NQDI-1 treatment as compared with the tIRI group (179 ± 21.2 vs. 123 ± 17.3, *p*-value = 0.0021) (Figure 2b). Contralateral testes showed normal GSH concentrations without significant variations (sham = 151 ± 27.2, tIRI = 154 ± 9.73 and NQDI1 = 164 ± 7.98, *p*-value > 0.05).

High MDA concentrations, a lipid peroxidation marker, were measured in ipsilateral testes subjected to tIRI as compared to sham (6.04 ± 2.42 vs. 3.10 ± 0.85, *p*-value = 0.0039) (Figure 2c). The NQDI-1-treated rats showed sham levels of MDA that were significantly lower than the tIRI-subjected testes (3.51 ± 0.71 vs. 6.04 ± 2.42, *p*-value = 0.0109). The contralateral testes showed no significant changes in MDA concentrations in all three animal groups (sham = 3.81 ± 0.8, tIRI = 2.72 ± 0.89 and NQDI-1 = 1.98 ± 0.55, *p*-value > 0.05).

**Figure 3:**
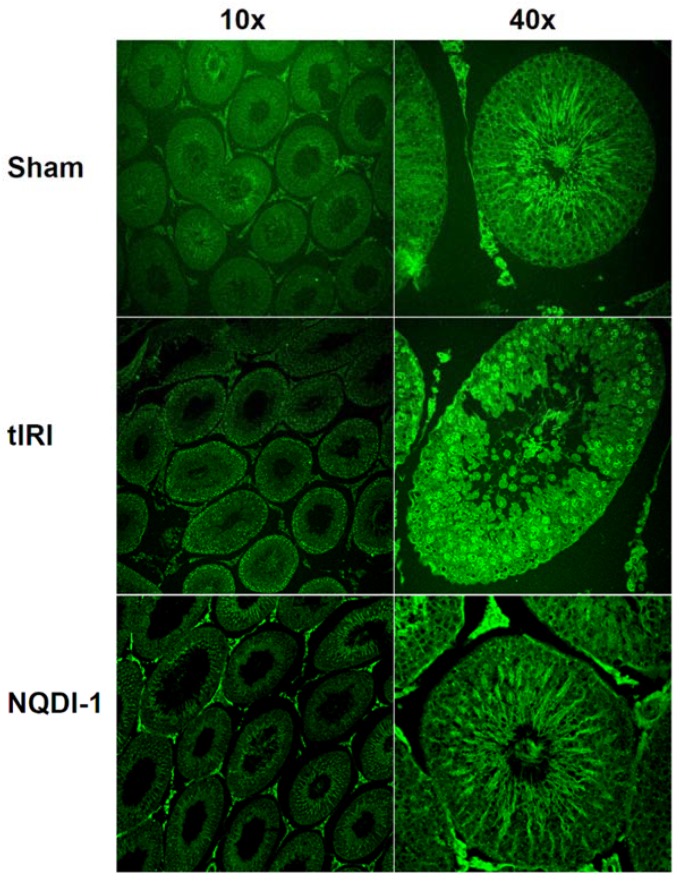
NQDI-1 prevents DNA strand breaks. Oxidative DNA strand breaks were assessed by counting TUNEL positive nuclei from 55 STs in testicular tissue sections/animal group. The STs of tIRI-subjected testes showed an increased number of fluorescently labelled free DNA 3′ ends in comparison to sham and NQDI-1-treated rats (*p*-value < 0.0001). Contralateral testes showed no significant difference between the three animal groups (*p*-value > 0.05). NQDI-1 (10 mg/kg) was i.p. injected 30 min post ischemia. Fluorescently stained tissue sections were visualized using the LSM700 confocal microscope. Images were taken at 10× and 40× magnification with a scale bar of 50 μm.

Oxidative DNA strand breaks were assessed by counting Terminal deoxynucleotidyl transferase dUTP Nick End Labeling (TUNEL) positive nuclei (Figure 2d and Figure 3). The tIRI-I-subjected testes exhibited a significant increase in the number of TUNEL positive nuclei compared to sham (87 ± 24 vs. 0.47 ± 1.7, *p*-value < 0.0001) and NQDI-1-treated group compared to tIRI (87 ± 24 vs. 3.8 ± 7.9, *p*-value < 0.0001). All contralateral testes had a sham like number of TUNEL positive nuclei with no significant difference (*p*-value < 0.05).

### 2.3. NQDI-1 Prevents Germ Cell Apoptosis

To detect the induction of tIRI-induced GCA and the influence of ASK1 signaling, the relative mRNA expression of pro-apoptosis and anti-apoptosis genes, caspase 3 activity and the activation of the ASK1/JNK/p38/survivin apoptosis pathway were measured. The relative mRNA expression was measured and calculated for the pro-apoptosis genes: *Bax, Bid* and *Bad* and for the anti-apoptosis genes: *Bcl2* and *Birc5* (survivin encoding gene) (Table 1). The relative mRNA expression of the pro-apoptosis genes *Bax, Bid,* and *Bad* was significantly upregulated in the tIRI group compared with sham (*p*-value < 0.05), which was normalized upon NQDI-1 treatment. In contrast, the relative mRNA expression of the anti-apoptosis genes *Bcl2* and *Birc5* (survivin) was suppressed (*p*-value < 0.05), but was restored after NQDI-1 treatment (*p*-value < 0.05). The *Bax* to *Bcl2* ratio showed a significant increase in the tIRI group compared to sham and NQDI-1-treated rats (*p*-value > 0.05). Contralateral testes revealed no significant differences in the relative mRNA expression of all genes among the three experimental groups (*p*-value > 0.05).

**Figure 4:**
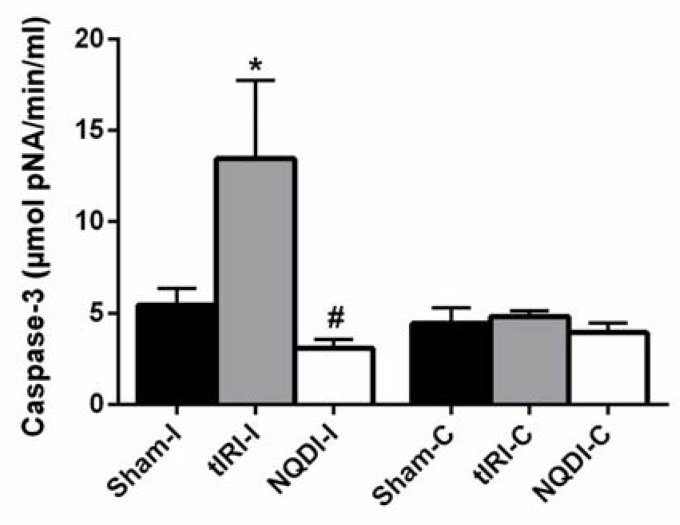
NQDI-1 attenuates Caspase 3 Activity. Utilizing a biochemical assay, tIRI-induced Caspase 3 activity was normalized by NQDI-1 treatment and similar to sham levels (*p*-value < 0.0001). Contralateral testes showed no significant difference between the three animal groups (*p*-value > 0.05). NQDI-1 (10 mg/kg) was i.p. injected 30 min post ischemia. Data are presented as mean ± SD (*n* = 6/group). * tIRI compared to sham and # NQDI-1 compared to tIRI. I = Ipsilateral and C = Contralateral.

The ipsilateral testes of tIRI-subjected rats revealed raised caspase 3 activity in comparison to sham (13.5 ± 4.29 vs. 5.47 ± 0.90, *p*-value < 0.0001) (Figure 4). The NQDI-1 treated rats showed sham-like levels of caspase 3 activity in comparison to tIRI rats (3.11 ± 0.47 vs. 13.5 ± 4.29, *p*-value < 0.0001). There was no significant difference in the caspase 3 activity among the contralateral testes (Sham = 4.46 ± 0.87, tIRI = 4.85 ± 0.31, and NQDI-1 = 3.96 ± 0.53, *p*-value > 0.05).

**Figure 5:**
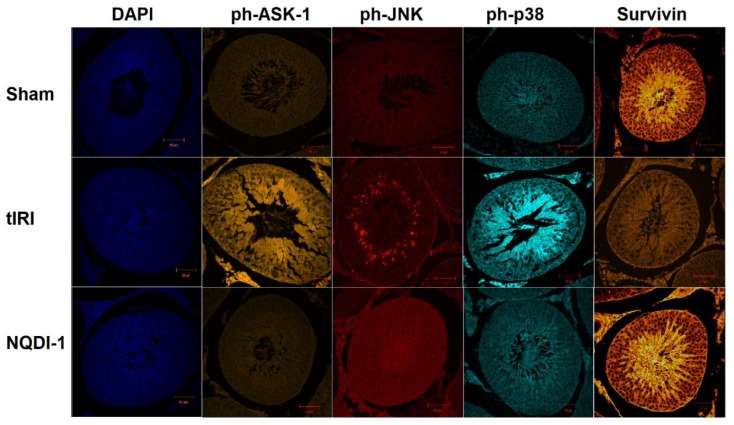
Immunofluorescence (IF) detection of the ph-ASK-1/ph-JNK/ph-p38/survivin apoptosis signaling pathway. The immuno-expression of the pro-apoptosis kinases ph-ASK-1/ph-JNK/ph-p38 was increased in spermatocytes in the ipsilateral testes-subjected to tIRI than in sham and after NQDI-1 treatment (*p*-value < 0.0001). The immune-expression of the IAP survivin was localized to spermatids and spermatozoa, however, it was significantly decreased in the tIRI-I group compared to sham and after NQDI-1 treatment (*p*-value < 0.0001). Contralateral testes showed no significant difference between the three animal groups (*p*-value > 0.05). NQDI-1 (10 mg/kg) was i.p. injected 30 min post ischemia. DAPI staining (blue) was carried out for all three animal groups. Fluorescently stained tissue sections (4 μm) were visualized using the LSM700 confocal microscope. Images were taken at 40× magnification with a scale bar of 50 μm.

Protein expression of phosphorylated ASK-1(ph-ASK-1), ph-JNK, ph-p38 and survivin were evaluated by IF staining (Figure 5). While the immunoexpression of phosphorylated ASK-1/JNK/p38 displayed ST localization to spermatocytes, survivin immunoexpression was localized to spermatids and spermatozoa. During tIRI, ph-ASK-1 showed high expression levels in comparison to sham (1255 ± 144 vs. 334 ± 42, *p*-value < 0.0001), while NQDI-1-treated rats showed low levels of ph-ASK-1 as compared to tIRI and similar to sham levels (348 ± 48 vs. 1255 ± 144, *p*-value < 0.0001). The immuno-expression of ph-JNK in the ipsilateral testes of tIRI testes was significantly increased compared to sham (1986 ± 173 vs. 731 ± 102, *p*-value < 0.0001), which was attenuated by NQDI-1 treatment (742 ± 24 vs. 1986 ± 173, *p*-value < 0.0001). A remarkable increase in the immuno-expression of ph-p38 was detected during tIRI compared with sham (1442 ± 383 vs. 743 ± 106, *p*-value < 0.0001), but was suppressed to sham levels upon NQDI-1 treatment (823 ± 165 vs. 1442 ± 383, *p*-value < 0.0001). As for survivin, significantly decreased immunoexpresion was exhibited during tIRI in comparison to sham group (427 ± 40 vs. 1299 ± 107, *p*-value < 0.0001) and to NQDI-1 group (427 ± 40 vs. 1305 ± 76, *p*-value < 0.0001). All contralateral testes displayed sham-like expression levels of ph-ASK-1, ph-JNK, ph-p38 and survivin (*p*-value > 0.05).

### 2.4. NQDI-1 Regulates the Expression of the Trx System

**Figure 6:**
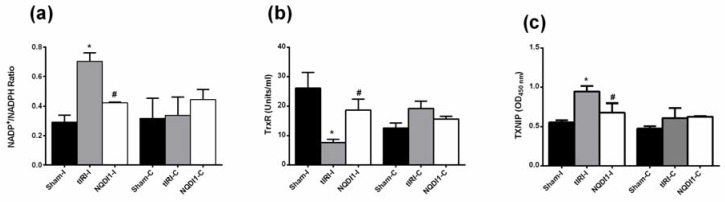
NQDI-1 regulates the expression of the Trx system. In the ipsilateral testes of tIRI-subjected rats, elevated levels of (**a**) NADP^+^/NADH ratio and (**b**) TXNIP were measured by a colorimetric assay and ELISA, respectively, however, Low (**c**) TrxR activity was measured using a kinetic assay in comparison to the sham group. Treatment with NQDI-1 reverted the levels of NADP^+^/NADH ratio, TXNIP and TrxR to sham levels. Contralateral testes showed no significant difference between the three animal groups (*p*-value > 0.05). NQDI-1 (10 mg/kg) was i.p. injected 30 min post ischemia. Data are presented as mean ± SD (*n* = 6/group). * tIRI compared to sham and # NQDI-1 compared to tIRI. I = Ipsilateral and C = Contralateral.

**Figure 7:**
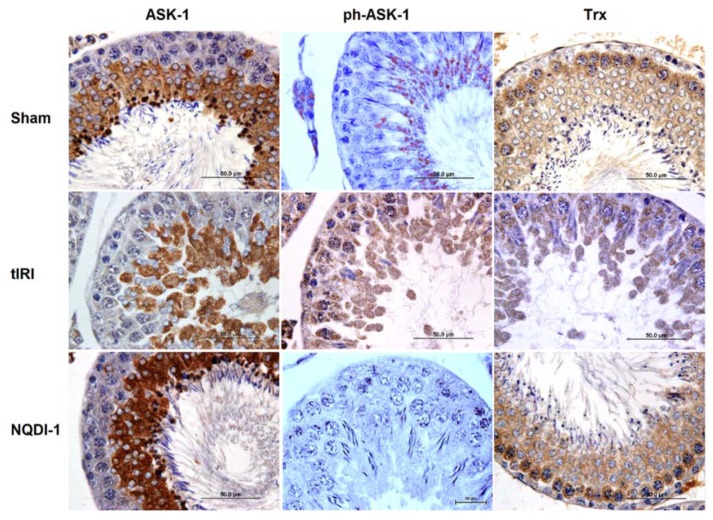
NQDI-1 modulates the expression of the ASK-1/Trx axis. The immunoexpression of the ASK-1, ph-ASK-1, and Trx were evaluated by IHC staining under light microscopy. Both Trx and ASK-1 showed reduced immunoreactivity in the tIRI-subjected testes in comparison with sham NQDI-1 treated groups. As for ph-ASK-1, it showed strong immunoreactivity in the tIRI-subjected testes in comparison with sham NQDI-1 treated groups. Contralateral testes showed no significant difference between the three animal groups. NQDI-1 (10 mg/kg) was injected i.p. 30 min post ischemia. Images were taken at 10× and 40× magnification with a scale bar of 50 μm.

In order to find out whether ASK1 regulates the Trx system, the expression of the Trx system components (NADPH, Trx, Trx Reductase, and TXNIP) were evaluated by realtime PCR, biochemical assays, and IHC staining (Table 2 and Figure 6 and Figure 7).

Ipsilateral testes of the tIRI group exhibited a significantly increased ratio of NADP^+^/NADPH compared with sham (0.70 ± 0.06 vs. 0.29 ± 0.05, *p*-value = 0.0013) (Figure 6a). Meanwhile, NQDI-1treated rats showed sham-like values in comparison to tIRI rats (0.42 ± 0.004, *p*-value = 0.0219). Contralateral testes showed a sham-like ratio of NADP to NADPH with no significant differences (sham = 0.32 ± 0.13, tIRI = 0.34 ± 0.12 and NQDI-1 = 0.44 ± 0.07, *p*-value > 0.05).

Across the ipsilateral testes, the tIRI group showed significantly lower activity of the TrxR enzyme as compared with sham levels (7.63 ± 1.08 vs. 26 ± 5.32, *p*-value < 0.0001) (Figure 6b). After NQDI-1 treatment, there was a steady recovery of the TrxR enzyme activity in comparison to tIRI (19 ± 3.6 vs. 7.63 ± 1.08, *p*-value = 0.0036). However, there was no significant differences in the TrxR enzyme activity among the contralateral right testes (Sham = 13 ± 1.7, tIRI = 19 ± 2.3 and NQDI-1 = 16 ± 0.95, *p*-value > 0.05).

Protein levels of TXNIP in the ipsilateral testes were significantly higher in the tIRI-subjected testes in comparison to sham (0.94 ± 0.071 vs. 0.56 ± 0.026, *p*-value < 0.0001), while it was reduced to sham levels after NQDI-1 treatment (0.68 ± 0.031, *p*-value = 0.0004) (Figure 6c). Contralateral testes from the three groups did show any changes in the TXNIP expression (sham = 0.48 ± 0.03, tIRI = 0.61 ± 0.13, and NQDI-1 = 0.63 ± 0.01, *p*-value > 0.05).

A significant decrease in the relative mRNA expression of *Txn1, Txn2, Txnrd1* and *Txnrd2* (*p*-value < 0.05) was calculated in the ipsilateral testes of the tIRI-subjected rats compared with sham rats (Table 2). The NQDI-1 treated rats had close to sham levels of *Txn* and *Txnrd* relative mRNA expression (*p*-value < 0.05). In contrast, the relative mRNA expression of *Txnip* in the tIRI-subjected rats was elevated compared to sham, which was normalized in NQDI-1 treated rats (*p*-value < 0.05). Contralateral testes revealed no significant differences in the relative mRNA expression of all genes among the three experimental groups (*p*-value > 0.05).

Protein expression of ASK-1, ph-ASK-1 and Trx were evaluated by IHC staining, which showed that ASK-1 expression was mainly expressed by spermatocytes, while Trx expression was throughout the ST (Figure 7). The immunoexpression of total ASK1 protein in the sham and NQDI-1 ipsilateral testes was at basal levels (5487 ± 1426, and 5513 ± 1977, respectively). However, the tIRI testes demonstrated lower than basal total ASK1 immunoexpression (1061 ± 959) in comparison to sham (*p*-value < 0.0001) and following NQDI-1 treatment (*p*-value < 0.0001). During tIRI, active ph-ASK1 had high expression levels in comparison to sham (15,746 ± 5144 vs. 2217 ± 1195, *p*-value < 0.0001), while NQDI-1-treated rats showed low levels of ph-ASK1 as compared to tIRI (2678 ± 748 vs. 15,746 ± 5144, *p*-value < 0.0001). On the other hand, the immuno-expression of Trx revealed decreased intensity in the tIRI group compared with sham (2036 ± 763 vs. 16,835 ± 4513, *p*-value < 0.0001), which was normalized after NQDI-1 treatment (15,427 ± 2973, *p*-value < 0.0001 compared to tIRI). Contralateral testes, on the other hand, did not show any noticeable changes in the immunoexpression of ASK-1, ph-ASK-1, and Trx (*p*-value > 0.05).

## 3. Discussion

The Trx system is a prevailing antioxidant system that is involved in redox regulation and is sensitive to high ROS production [11]. Earlier evidence indicates that Trx provides cytoprotection against OS, offers redox control and regulates cell proliferation [11,12]. Thus, disrupted Trx system expression would strongly promote apoptosis [13]. During OS, Trx is post-translationally modified at Cys-32 and Cys-35 causing its inactivation [14]. The intra-disulfide bond between Cys-32 and Cys-35 of the oxidized form releases Trx from ASK-1, thus, activating signal transduction of downstream MAPKs [14,15]. It was also reported that Trx overexpression in vivo reduced ASK-1 levels, inhibited ASK-1-induced JNK activity and that Trx-ASK-1 dissociation was found to be ROS dependent [14]. Furthermore, BAECs overexpressing ASK-1 confirmed the activation of the ASK-1/JNK/caspase 3 pathway. This provided a great insight on the interplay between Trx/ASK-1/JNK in the OS-induced GCA in our tIRI model. In addition, there is experimental evidence that p38 becomes activated post Trx-ASK-1 dissociation as a consequence of ROS-induced OS [11]. Inhibition of reduced Trx by rotenone (ROT), a ROS inducer, in hepatocytes led to Trx-ASK1 complex dissociation and inducing the p38 pathway, which was reverted by N-acetyl cysteine treatment [16]. This suggests that reduced Trx conveys indirect protective function by modulating p38 and ASK-1 signaling factors and hence regulate their stress responsive pathways. Also, ASK-1 deficient thymocytes prevented apoptosis even in the presence of a stress stimulus by repressing the JNK/p38 signaling pathway [17]. Stress preconditioned neuroblastoma SH-SY5Y cells had escalated the synthesis of reduced Trx, active TrxR, and Bcl-2, a pro-apoptosis protein. Meanwhile, the cells subjected to 24 h stress exhibited apoptosis induction as well as lipid peroxidation. Cell transfection with a *Trx* antisense drastically reduced Bcl-2 levels. Transcription of *Trx* is regulated by cGMP via interaction with the AP-1 binding site in the *Trx* and *c-Jun* promoter regions, suggesting a sequential activation route for apoptosis induction mediated by OS [15]. Based on serum deprivation and methyl-4-phenylpyridinum (MPP^+^) induced apoptosis model on human SH-SY5Y post OS, incubation with Trx showed lack of cytosolic cytochrome *c* with remarkable increase in *Bcl-2* expression. Whereas the absence of Trx strictly increased cytochrome *c* release from the mitochondria [15]. Protein kinases within the MAPK superfamily were recognized to coordinate various stages of cell division throughout spermatogenesis for proper fertility. In this instance, JNK and p38 MAPKs play vital roles [18]. The inhibition of p38 in rat models using cadmium diminished germ cell loss in the seminiferous epithelium [19]. Recently, it was demonstrated that ongoing GCA revealed activated p38 that disrupted the blood testis barrier [20]. The same group also showed high expression levels of p38 in the testes of Eriocheir sinensis (Chinese mitten crabs) during several spermatogenic stages. It was also found that p38 was involved in the regulation of cell survival during spermatogenic proliferation and to be a negative regulator to sperm motility in mouse testis [21]. Other studies linked another member of ASK-1 downstream kinases, JNK, to spermatogenesis. It is found that JNK was also expressed in the testis to regulate Sertoli cell tight junction barrier in addition to GCA [18,22]. Moreover, inhibition of JNK and p38 signaling using their selective inhibitors had a positive effect on sperm capacitation, sperm rate, oxidative DNA damage, caspase-3/7 activation and apoptosis [23,24]. Our results clearly establish the vital role of the Trx system in the pathogenesis of tIRI and its cross talk with the ASK-1/JNK/p38 apoptosis signaling pathway during testicular OS, as evidenced by the use of NQDI-1.

It was established that the antioxidant reduced state of Trx is due to the TrxR enzyme activity and the critical role of NADP^+^/ NADPH [11]. Thus, one can suggest that the importance of Trx as an oxidizing equivalent in apoptosis regulation is strongly related to the ability of TrxRs as reducing equivalents to protect the cells from oxidative injuries. As such, the inhibition of any member of the Trx system is pathologic [25]. For example, inhibition of TrxR gene in Saccharomyces cerevisiae sufficiently decreased the levels of reduced Trx [26]. Also, high Trx expression was associated with high TrxR activity and elevated NADPH levels. This overexpression was transcriptionally regulated as indicated by the upregulation of the genes encoding Trx and TrxR: *Txn1*, *Txn2* & *Txnrd1, Txnrd2,* respectively. Sperm morphology, motility and viability were also correlated with increased apoptosis triggered by high ROS levels and p53, which is negatively correlated with TrxR enzyme activity [27]. Thus, TrxR and its active substrate Trx serve as cytoprotectors against OS and maintain the redox state in spermatozoa during normal spermatogenesis [28]. However, accumulation of oxidized Trx and inhibition of p53 activity is directly related to decreased TrxR activity thus, strongly suggesting the negative correlation between p53 and TrxR with regard to apoptosis induction [29]. This supports our findings that the relative mRNA expression of *Txn1*, *Txn2, Txnrd1,* and *Txnrd2* were downregulated subsequent to the tIRI. As an electron carrier, NADPH and its oxidized couple NADP^+^ are critical in anabolic redox reactions and maintenance of cellular redox balance [30]. Thus, their physiological concentrations and ratio are vital for the actual redox potential and must be conserved [31]. Studies on post ischemic heart demonstrated that depletion of NADPH resulted in loss of the eNOS function and failure of heme and tetrahydrobiopterin (BH_4_) recycling, while NADPH supplementation enhanced cardiac contractile restoration and decreased infarction [32]. This is in agreement with our findings whereby a decreased NADP^+^/NADPH ratio and suppressed TrxR activity were measured following tIRI. Inhibition of the above modulations by NQDI-1 further proves the regulation of the Trx system by ASK-1 signaling.

A natural and important intracellular negative regulator of Trx is TXNIP, which has reducing capabilities [33]. The importance of TXNIP is its ability to bind all Trx isoforms through disulfide linkages and it serves as apoptosis mediator in pathological conditions including IRI by controlling oxidative metabolism and *OS*-induced apoptosis [34,35]. Increased *Txnip* mRNA expression by 2.06-folds and TXNIP immuno-expression were detected in the cytoplasm of neurons post focal cerebral ischemia that was associated with severe brain tissue damage [36]. Furthermore, a clear translocation of TXNIP protein was demonstrated post OS to the mitochondria, where it is found to be linked to Trx2 [37]. The interaction between TXNIP and Trx2 facilitate the oxidation of Trx2, its dissociation from its bound ASK-1 under physiological conditions and further activates the ASK-1 downstream apoptotic pathway [38]. This greatly supports our findings in terms of the transcriptional and post-transcriptional upregulation of TXNIP in response to testicular OS leading to GCA, which was attenuated by NQDI-1. Thus, we can suggest that TXNIP is a modulator of oxidative metabolism pathway and the related ASK-1 apoptosis pathway of tIRI.

Under extreme oxidative stress conditions, ASK-1 activates JNK, which regulates the expression of apoptosis related proteins. In response to a death signal, the pro-apoptosis proteins BID and BAD are activated transcriptionally and/or post-translationally modified. The ‘‘sensitizer’’ BAD protein binds to the anti-apoptotic protein Bcl-2, releasing the ‘‘activator’’ BID, which in turn activates the downstream proteins Bax and BAK to initiate apoptosis. The *Bax* to *Bcl-2* ratio is considered as a cellular rheostat for cell survival or death. Reoxygenation of a torsed testis upregulates Bax expression 24 h post detorsion, causes cytochrome *c* release, initiates caspase cascade and leads to DNA degradation, but no apparent increase in Bcl-2 was measured [39]. Other studies also pointed that apoptosis of spermatogenic cells is under the command of the *Bax/Bcl-2* ratio and thus can be considered as a regulator of spermatogenic cell apoptosis [40,41]. A recent study showed that curcumin treatment inhibited OS and attenuated the *Bax* to *Bcl-2*-mediated cell death pathway in the testes of diabetic rats [42]. It was also established that survivin, an inhibitor of apoptosis (IAP), is a powerful inhibitor of caspase-3-induced cell death, in a specialized IAP-caspase-3 complex. In in vitro studies, cell death was evaluated after transfecting two cell lines one with caspase 3 containing plasmid only and the second with IAPs (*Tiap* or *Xiap*). The murine TIAP was used based on its 84% sequence homology to human survivin, as well as its high expression in the testis [43]. Cells expressing caspase 3 only underwent apoptosis in a higher rate than those expressing *Tiap or Xiap. Thus confirming that* caspase 3 was indeed responsible for cell execution after cellular injury. The above studies are supportive of our results that ASK-1/JNK signaling controls the expression of pro- and anti-Apoptosis genes and caspase 3 activity to modulate GCA.

Germ line deterioration is attributed to DNA fragmentation during normal spermatogenesis as well as due to pathological oxidative conditions. However, the majority of DNA damage in spermatozoa is due to excessive exposure to ROS, leading to oxidative attack and insufficient antioxidants such as SOD [44]. Transcriptional activation of either form of the three SODs is linked to the regulatory DNA elements found in the proximal promoter regions of the *sod* genes that provide binding sites for transcription factors (TFs), including AP-1 and p53 [45]. Active JNK phosphorylates c-Jun, a member of the AP-1 family, and promote the binding activity of AP-1 to its DNA element and hence the transcription of many genes [46]. Since *Trx* has an AP-1 DNA element its promoter region, AP-1 and c-Jun are thought to be involved in redox regulation that modulate cellular processes post oxidative damage [47]. This suggests a link between the ASK-1/JNK and the Trx system axes. SOD2 deficient mice showed boosted transcriptional activity of AP-1 and p53, suggesting that SODs are under the command of JNK/p38 signaling pathway, thereby, linking SODs to mitochondrial apoptosis [48]. Unfortunately, our findings regarding the SOD activity are inconclusive, thus, we are unable to confirm ASK-1 regulation of SOD activity in this study.

Although the exact mechanism of the pathophysiology of tIRI is not completely resolved, the results from this study suggest an interplay between cellular redox homeostasis, antioxidant systems and male germ cell apoptosis signaling to partly explain the tIRI-induced oxidative damages and germ cell death mechanism. In that context, the action mechanism of ASK-1 signaling clearly involves the regulation of the cellular antioxidant Trx system as demonstrated by the use of NQDI-1. This unique aspect of the role of the ASK-1/Trx axis in tIRI makes NQDI-1 a plausible candidate as an adjuvant therapy prior to testicular detorsion and a double edged tool that can enhance the cellular antioxidant system and simultaneously inhibit GCA signaling in order to avoid future male infertility issues. However, further experiments utilizing antisense technology and other inhibitors targeting the Trx system are in need to further strengthen our findings.

## 4. Materials and Methods

### 4.1. Ethics Statement

Male Sprague-Dawley (SD) rats (Charles River, Waltham, MA, USA) were handled following the guidelines set by the ethics committee at Kuwait University. The approved protocol (YM 09/17) is in agreement with the International Council for Laboratory Animal Sciences (ICLAS). The rats were housed in a controlled environment with 12-h dark/12-h light cycle at 25 °C and supplied with standard commercial diet and water at all times.

### 4.2. Drug

Ethyl 2,7-dioxo-2,7-dihydro-3*H*-naphtho[1,2,3-*de*]quinoline-1-carboxylate (NQDI-1) was purchased from Sigma-Aldrich (St. Louis, MO, USA). The drug preparation and concentration used were as reported previously [9,49]. NQDI-1 was dissolved in DMSO (Sigma-Aldrich; St. Louis, MO, USA) at a stock concentration of 25 mg/mL and administered intraperitoneally (i.p.) at 10 mg/kg.

### 4.3. Animals and Surgical Procedure

Male SD rats (*n* = 36, 8 weeks old, 250–300 g) were divided equally into three experimental groups: sham, unilateral tIRI only, and tIRI + NQDI-1. The rats were anesthetized with 50 mg/kg ketamine (Hikma Pharmaceuticals, Amman, Jordan) and 2 mg/kg xylazine (Bayer GmbH, Leverkusen, Germany) as an i.p. injection. The tIRI model was previously reported [50]. During the Sham procedure, an incision at the left ilioinguinal side was made and the left testis was exposed for 1 h before returning it into its scrotal sac followed by wound closing with surgical clips. Sham rats were sacrificed after 4 h. In the unilateral tIRI only group, testicular ischemia was induced by clamping the spermatic cord and artery of the left testis with a 700 g pressure straight bulldog clamp for 1 h (Roboz Surgical Instruments Co., Gaithersburg, MD, USA). An i.p. injection of 250 µL DMSO was administered 30 min prior to reperfusion. One-hour post ischemia, the clamp was removed and the testis was allowed to reperfuse for 4 h before animal sacrifice. The NQDI-1 group followed the same procedure as the unilateral tIRI only, but NQDI-1 (10 mg/kg) was i.p. injected instead of DMSO. The right contralateral testis in all groups was used as an internal positive control.

### 4.4. RNA Isolation, Reverse Transcription (RT) and Real-Time PCR

TRIzol (Invitrogen, Carlsbad, CA, USA) was used to purify total RNA from frozen testes following the manufacturer’s instructions. Total RNA concentration was measured spectrophotometrically at 230, 260 and 280 nm and samples were stored at −80 °C. Synthesis of cDNA by RT from 2 μg of purified RNA was performed using the high-capacity cDNA reverse transcription kit (Thermo Fisher Scientific, Waltham, MA, USA) following the manufacturer’s protocol. For real-time PCR reactions, pre-designed and validated TaqMan® gene expression assays (Thermo Fisher Scientific, Waltham, MA, USA) were used to quantify the relative gene expression for Trx-1 (*Txn1*, Rn00587437_m1), Trx-2 (*Txn2*, Rn00584162_g1), Trx interacting protein (*Txnip*, Rn01533891_g1), Trx reductase-1 (*Txnrd1*, Rn01503798_m1), Trx reductase-2 (*Txnrd2*, Rn00574868_m1), B-cell lymphoma 2 (*Bcl-2*, Rn99999125_m1), Bcl-2 associated X (*Bax*, Rn01480161_g1), Baculoviral IAP repeat containing 5 (*Birc5*, Rn00574012_m1), BH3 interacting domain (*Bid*, Rn01459517_m1), Bcl-2 associated agonist of cell death (*Bad*, Rn00575519_m1) and β-actin as an endogenous control (*Actb*, Rn00667869_m1). The real-time PCR reaction mix contained a 2× Taqman universal PCR master mix (Thermo Fisher Scientific, Waltham, MA, USA), cDNA template, 20× Taqman assay and the final volume of 20 μL was completed with nuclease-free water. Reaction mixtures were prepared in a 96-well reaction plate and placed in the 7500 Sequence Detection System (Applied Biosystems, Foster City, CA, USA). Cycling parameters recommended by the manufacturer were used. The relative mRNA expression was calculated using the 2^−ΔΔCt^ method on the basis of mean Ct values [51]. As such, the gene expression in the sham group was used as a calibrator (set as 1).

### 4.5. Biochemical Analyses

RIPA buffer (Thermo Fisher Scientific, Waltham, MA, USA) was used to isolate total proteins from frozen testicular tissues. Total protein concentration was quantitated by the Ultrospec 2100-Pro Biochrom Spectrophotometer (Biochrom, Cambridge, UK) at 595 nm using a Bovine serum albumin standard curve. Stock protein samples were aliquoted and stored at −80 °C. Working concentrations of protein samples were standardized for each biochemical assay.

Superoxide dismutase (SOD) activity: The colorimetric assay SOD determination Kit (Sigma-Aldrich, St. Louis, MO, USA) was carried out following the manufacturer’s protocol. The SOD activity was calculated as a percentage (%).

Caspase-3 activity: Caspase-3 enzyme activity was determined using a Caspase 3 assay kit following the manufacturer’s recommendations (Sigma Aldrich, St. Louis, MO, USA). A pNA calibration curve was used to calculate caspase 3 activity as a µmol/min/mL sample.

Thioredoxin Reductase (TrxR) activity: The TrxR assay kit (Sigma-Aldrich, St. Louis, MO, USA) is a kinetic colorimetric assay designed in a 96-well plate. The TrxR enzymatic activity was measured following the manufacturer’s protocol and presented as Unit/mL sample.

NADP^+^/NADPH Quantification: The NADP/NADPH quantification kit was used following the manufacturer’s recommendations (Sigma-Aldrich, St. Louis, MO, USA). The assay is designed to quantify NADP^+^, NADPH, and their ratio (but not NAD^+^ or NADH) without the need to purify them.

Malondialdehyde (MDA) Assay: The BIOXYTECH® MDA-586 kit (Oxis Research, Portland, OR, USA) was used for colorimetric quantification of MDA, a lipid peroxidation marker. MDA concentration (μM) was measured at 586 nm and calculated using an MDA standard curve.

Glutathione (GSH): The Glutathione assay kit (Sigma-Aldrich, St. Louis, MO, USA) was used to measure total GSH concentration in the tissue extracts following the manufacturer’s protocol. The concentration of GSH was calculated as nmoles GSH/mL of the sample.

Enzyme-linked immunosorbent assay (ELISA) of TXNIP: The rat TXNIP ELISA kit was used following the manufacturer’s protocol (Bio-Connect Diagnostics, Huissen, The Netherlands). TXNIP levels were detected at 450 nm using the CLARIOstar micro-plate reader (BMG Labtech, Ortenberg, Germany).

### 4.6. Histological Examination

Tissue Processing: Harvested testes were preserved in Bouin’s fixative, hydrated, and embedded in paraffin. Tissue sections (4 µm) were used for hematoxylin and eosin (H&E) staining, immunohistochemistry staining (IHC) and immunofluorescence (IF) staining.

H&E staining: Slides from all testes were stained with H&E following the rehydration and dehydration steps. Each section was mounted with DPX and a coverslip. Ten seminiferous tubules (STs)/group were examined under light microscopy (40×) to evaluate spermatogenesis on the basis of the Johnsen score to estimate the maturation level in each ST using the scale of 1–10 [52].

IHC staining: Testicular paraffin sections (4 µm) were dewaxed and incubated in a blocking solution (Invitrogen, Frederick, MD, USA) post rehydration and dehydration steps. Section were then incubated overnight at 4 °C with primary antibodies (1:50 dilution) for ASK-1 (Bioss antibodies, Woburn, MA, USA), ph-ASK1 (Bioss Antibodies, Woburn, MA, USA) and Trx antibody (Abcam, Cambridge, UK). PBS-washed slides were then incubated with a broad-spectrum secondary antibody (60:40) (Thermo Fisher Scientific, Waltham, MA, USA) and antibody-antigen interaction was detected with HRP-Streptavidin (Thermo Fisher Scientific, Waltham, MA, USA). The sections were then stained with the chromogen 3,3′-diaminobenzidine (DAB) using the Impact DAB kit (Vector Laboratories, Burlingame, CA, USA). Finally, slides were counterstained with hematoxylin and mounted with DPX. The immunolabeling intensity was assessed by measuring the color intensity (Sum (Area) (pixel2)) using the Olympus DP 71 camera and Cell Sens Dimension Software (Olympus, Tokyo, Japan) using 2 slides/group.

IF staining: Slides were dewaxed and rehydrated prior to antigen microwave retrieval in citrate buffer (0.01 M, pH 6). Blocked slides were then incubated overnight with primary antibodies for p-ASK1 (1:25 dilutions; Bioss Antibodies, Woburn, MA, USA), p-JNK (1:100 dilutions; Santa Cruz Biotechnology, Dallas, TX, USA), p-p38 (1:25 dilutions; Cell Signaling Technology, Danvers, MA, USA) and survivin (1:50 dilution; Santa Cruz Biotechnology, Dallas, TX, USA). This was followed by incubation with Goat anti-mouse IgG (H + L) highly cross-adsorbed secondary antibody, Alexa Fluor 488 and Alexa Fluor 555 (1:5000 dilutions; Thermo Fisher Scientific, Waltham, MA, USA). Finally, slides were mounted with DAPI, covered and sealed. Fluorescently stained slides were analyzed using the LSM700 confocal microscope (Carl Zeiss Micro-Imaging GmbH, Jena, Germany). The IF intensity for each antibody was measured in 15 STs/group utilizing the ZEN2 (blue edition) software.

Terminal 2-deoxyuridine 5-triphosphate nick end-labeling assay (TUNEL): The in Situ Cell Death Detection Kit, Fluorescein kit (Roche-Diagnostics, Mannheim, Germany) was used to evaluate oxidative DNA strand breaks by detecting the free 3′-OH termini following the manufacturer’s protocol. The slides were analyzed with the LSM 700 confocal laser scanning microscope (Carl Zeiss Micro-Imaging GmbH, Jena, Germany). DNA strand breaks, represented by TUNEL positive nuclei, was evaluated in more than100 randomly selected STs/group. UNEL positive cells were counted using 40x magnification images, while 10× magnified images were captured for presentation only.

### 4.7. Statistical Analysis

All data were subjected to statistical analysis using the GraphPad Prism software v6.0 (GraphPad Software Inc., San Diego, CA, USA). The numerical data sets were assessed by the One-way Analysis of Variance (ANOVA). The mean values were further assessed by the Holm-Sidak’s multiple comparison test between each pair of experimental groups. Data are presented as mean ± standard deviation (SD). The personal bias was excluded following blinded manner analysis throughout experimental procedures. The *p*-value < 0.05 was considered statistically significant.

## Figures and Tables

**Table 1:** Relative mRNA expression of apoptosis-related genes calculated by the 2^−ΔΔCT^ formula.

Gene Name	Sham	tIRI	* *p*-Value	NQDI-1 ^1^	^#^*p*-Value
Pro-Apoptosis Genes					
*Bax* I	1.0 ± 0.0	2.4 ± 0.8	<0.0001	0.8 ± 0.1	<0.0001
C	1.0 ± 0.0	0.9 ± 0.4	>0.9999	0.8 ± 0.3	0.9957
*Bid* I	1.0 ± 0.0	13 ± 1.9	<0.0001	1.2 ± 0.6	<0.0001
C	1.0 ± 0.0	1.1 ± 0.1	>0.9999	1.0 ± 0.1	>0.9999
*Bad* I	1.0 ± 0.0	7.6 ± 1.5	<0.0001	0.9 ± 0.3	<0.0001
C	1.0 ± 0.0	0.9 ± 0.1	>0.9999	0.9 ± 0.3	>0.9999
Anti-Apoptosis Genes					
*Bcl-2* I	1.0 ± 0.0	0.2 ± 0.01	0.003	1.1 ± 0.2	0.001
C	1.0 ± 0.0	1.1 ± 0.5	>0.9999	0.9 ± 0.7	0.9996
*Birc5* I	1.0 ± 0.0	0.2 ± 0.04	<0.0001	0.9 ± 0.2	<0.0001
(Survivin) C	1.0 ± 0.0	1.1 ± 0.2	0.5772	1.0 ± 0.1	0.7213
*Bax/Bcl-2* I	1.0 ± 0.0	17 ± 1.6	0.0012	1.3 ± 0.4	0.0014
*Ratio* C	1.0 ± 0.0	0.9 ± 0.1	>0.9999	1.6 ± 0.8	>0.9999

^1^ Rats received an i.p. injection of NQDI-1 (10 mg/kg) 30 min prior to reperfusion. Data analysis was determined by the one way analysis of variance (ANOVA) accompanied by the Holms-Sidak multiple comparisons test. Relative mRNA expression is presented as mean ± SD (*n* = 6). * tIRI compared to sham; ^#^ NQDI-1 compared to tIRI. I = ipsilateral; C = contralateral.

**Table 2:** Relative mRNA expression of the Trx system genes calculated by the 2^-ΔΔCT^ formula.

Gene Name	Sham	tIRI	* *p*-Value	NQDI-1 ^1^	^#^*p*-Value
*Txn1* IC	1.0 ± 0.00	0.4 ± 0.09	<0.0001	0.0 ± 0.21	<0.0001
	1.0 ± 0.00	0.8 ± 0.15	0.1827	0.8 ± 0.24	0.9927
*Txn2* IC	1.0 ± 0.00	0.2 ± 0.04	<0.0001	0.9 ± 0.15	<0.0001
	1.0 ± 0.00	0.8 ± 0.15	0.0973	0.8 ± 0.24	0.9860
*Txnrd1* IC	1.0 ± 0.00	0.1 ± 0.16	<0.0001	0.9 ± 0.09	<0.0001
	1.0 ± 0.00	0.9 ± 0.08	0.0837	0.9 ± 0.09	>0.9999
*Txnrd2* IC	1.0 ± 0.00	0.2 ± 0.07	<0.0001	0.9 ± 0.22	<0.0001
	1.0 ± 0.00	1.0 ± 0.18	>0.9999	0.9 ± 0.22	0.9453
*Txnip* IC	1.0 ± 0.00	6.3 ± 1.10	<0.0001	1.1 ± 0.41	<0.0001
	1.0 ± 0.00	1.4 ± 0.93	0.7973	1.3 ± 0.43	0.9994

^1^ Rats received an i.p. injection of NQDI-1 (10 mg/kg) 30 min prior to reperfusion. Data analysis was determined by the one way analysis of variance (ANOVA) accompanied by the Holms-Sidak multiple comparisons test. Relative mRNA expression is presented as mean ± SD (*n* = 6). * tIRI compared to sham; ^#^ NQDI-1 compared to tIRI. I = ipsilateral; C = contralateral.

## Abbreviations

ANOVA	Analysis of Variance
AP-1	Activator Protein-1
ASK-1	Apoptosis Signal regulating Kinase-1
BAD	Bcl2 associated agonist of cell death
BAEC	Bovine Aorta Endothelial Cells
BAX	Bcl2 associated X
BCL-2	B-cell lymphoma 2
BID	BH3 interacting domain
Birc5	Baculoviral IAP repeat containing 5
BSA	Bovine Serum Albumin
cDNA	Complementary deoxyribonucleic acid
cGMP	Cyclic Guanosine mono phosphate
DAB	Chromogen 3,3′-diaminobenzidine
DAPI	4′,6 diamidino-2-phenyl indole
DNA	Deoxyribonucleic acid
DPX	Distyrene Plasticizer Xylene
ELISA	Enzyme-linked immunosorbent assay
GCA	Germ Cell Apoptosis
GSH	Glutathione
i.p.	Intraperitoneally
IAP	Inhibitor of Apoptosis
ICLAS	International Council for Laboratory Animal Sciences
JNK	c-jun N-terminal kinase
MAPK	Mitogen Activated Protein Kinase
MDA	Malondialdehyde
MPP^+^	1-methyl-4-phenylpyridinum
NADP+/NADPH	Nicotinamide adenine dinucleotide phosphate
NQDI-1	Ethyl 2,7-dioxo-2,7-dihydro-3*H*-naphtho[1,2,3-*de*]quinoline-1-carboxylate
OS	Oxidative Stress
PBS	Phosphate buffer solution
PCR	Polymerase Chain Reaction
ph-JNK	Phosphorylated JNK
ph-p38	Phosphorylated p38
RIPA	Radioimmunoprecipitation assay
RNA	Ribonucleic acid
ROS	Reactive Oxygen Species
RT	Reverse Transcription
SD	Sprague-Dawley
SD	Standard Deviation
SOD	Superoxide dismutase
ST	Seminiferous Tubule
TD	Testicular Detorsion
TFs	Transcription Factors
tIRI	Testicular Ischemia Reperfusion Injury
TRX	Thioredoxin
TrxR	Thioredoxin Reductase
TrxR-1	Thioredoxin Reductase-1
TrxR-2	Thioredoxin Reductase-2
TT	Testicular Torsion
TTD	Testicular Torsion and Detrosion
TUNEL	Terminal deoxynucleotidyl transferase dUTP Nick End Labeling
TXNIP	Thioredoxin Interacting Protein
